# Clinicopathological Characteristics of Inflammatory Myofibroblastic Tumor: A Single Center Retrospective Cohort Study

**DOI:** 10.1111/1759-7714.15496

**Published:** 2024-11-26

**Authors:** Xiaoyan Si, Shafei Wu, Ruie Feng, Mengzhao Wang, Hanping Wang, Xiaotong Zhang, Li Zhang, Kaifeng Xu

**Affiliations:** ^1^ Department of Pulmonary and Critical Care Medicine Peking Union Medical College Hospital, Chinese Academy of Medical Sciences, Peking Union Medical College Beijing China; ^2^ Department of Pathology Peking Union Medical College Hospital, Chinese Academy of Medical Sciences, Peking Union Medical College Beijing China

**Keywords:** anaplastic lymphoma kinase, chemotherapy, inflammatory myofibroblastic tumor, targeted therapy

## Abstract

**Background:**

Inflammatory myofibroblastic tumor (IMT) is a rare intermediate‐grade neoplasm. It presents a great challenge in diagnosis and treatment. This study aims to identify the clinicopathological characteristics of IMT.

**Methods:**

A retrospective study was conducted, enrolling patients with IMT at Peking Union Medical College Hospital from January 2013 to October 2023. Clinical information, treatments, and efficacy were analyzed.

**Results:**

A total of 72 patients were enrolled, including 38 men and 34 women, with a median age of 46.5 years. The most common primary site included the lung (*n* = 15, 20.8%), intestinal tract (*n* = 8, 11.1%), abdominal cavity (*n* = 7, 9.7%), and nasal sinus (*n* = 5, 6.9%). Thirty patients harbored anaplastic lymphoma kinase (ALK) fusion genes; Sixty‐five (90.3%) patients underwent surgical resection, and 11 of them had postoperative recurrence. Thirty patients received systemic therapy, including nonsteroidal anti‐inflammatory drugs (*n* = 1), steroids (*n* = 5), chemotherapy (*n* = 7), targeted therapy (*n* = 2), and immune checkpoint inhibitor (*n* = 1).

**Conclusions:**

The most common site of IMT is the lung. Surgery is the main treatment for IMT, and postoperative adjuvant therapy for ALK‐positive patients needs to be focused. The molecular testing is essential for all patients diagnosed with IMTs. Systemic treatment needs further research.

## Graphical abstract

The study was to summarize the clinicopathological features of inflammatory myofibroblastic tumors. Chest CT scans of most pulmonary IMT patients showed a well‐delineated solitary mass (Figures A–B). Only one patient had a ground glass nodule on the CT scan (Figure C). One case of advanced pulmonary IMT manifested as hilar and mediastinal lymph node metastasis. (Figure D) CT scan of the abdominal cavity, renal, hepatic, and nasal sinus IMT showed a solid mass with heterogeneous or homogeneous enhancement. (Figures E–H).

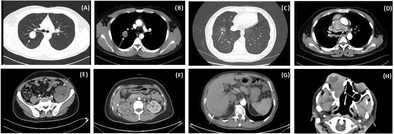

## 1 Introduction

Inflammatory myofibroblastic tumor (IMT) is a rare intermediate‐grade tumor that has the potential to be malignant, including invasion, metastasis, and recurrence. IMT is characterized histologically by the proliferation of fibroblasts and myofibroblasts, accompanied by chronic inflammatory infiltration by lymphocytes, plasma cells, eosinophils, and histiocytes. IMT can occur in the lung, neck, digestive tract, liver, urinary tract, uterus, and breast [1]. The main treatment for IMT is surgical resection [2]. However, some IMT cases have recurred after surgery, and treatment options are limited for patients with unresectable and/or advanced disease. In recent years, the application of next‐generation sequencing (NGS) in tumors has greatly promoted the genomic characterization of IMT [3]. This study aimed to summarize the clinical features and treatment of IMT. We present this article in accordance with the STROBE reporting checklist [4].

## 2 Methods

### 2.1 Study Design and Patient Selection

This study was conducted in a single‐center, observational, and retrospective cohort. The study was conducted by the Declaration of Helsinki (as revised in 2013). The study was approved by the Institutional Review Board of Peking Union Medical College Hospital (No. I‐23PJ1278), and individual consent for this retrospective analysis was waived. IMT comprises differentiated myofibroblastic spindle cells with numerous plasma cells and/or lymphocyte infiltrates according to the World Health Organization (WHO) classification, and pathological diagnosis should differentiate from various spindle cell tumors and reactive proliferative diseases, such as inflammatory fibroid polyp, nodular fasciitis, gastrointestinal stromal tumor, desmoid fibromatosis, and IgG4‐related disease. The patients diagnosed with IMT in Peking Union Medical College Hospital from January 2013 to October 2023 were searched in two databases: (1) The Department of Medical Records of Peking Union Medical College Hospital, and (2) the information system of the Department of Pathology of Peking Union Medical College Hospital. The patients searched above were further selected according to inclusion and exclusion criteria. The inclusion criteria were: (1) A histologically confirmed diagnosis of IMT by the Department of Pathology, Peking Union Medical College Hospital. The exclusion criteria were: (1) Combined with other malignant tumors; (2) lack of clinical information. The patient demographic and clinical characteristics were reviewed from medical records. The objective of the study was to assess the clinicopathological features and efficacy of treatment.

### 2.2 Sample Collection, Processing, and Sequencing

ALK immunohistochemical analysis (IHC) was routinely performed in IMT patients. NGS was performed for IMT patients with ALK‐negative if there were enough specimens within 3 years. Tumor DNA and RNA for targeted sequencing were obtained from FFPE tissue by the MagPure FFPE DNA/RNA LQ Kit (Magen, Guangzhou, China), following the manufacturer's instructions. The extracted DNA/RNA concentration was measured by the QuantiFluor dsDNA System and QuantiFluor RNA System (Promega, Madison, Wisconsin, USA). Sequencing was performed on each patient's tumor DNA using NGS targeting 40 cancer genes and assessing microsatellite instability (MSI) status (AmoyDx HANDLE Classic Panel). All included genes are listed in Table S1.

### 2.3 Response Evaluation

Tumor responses (CR, complete remission; PR, partial response; SD, stable disease; and PD, progressive disease) were evaluated according to the RECIST version 1.1. Disease control rate (DCR) was defined as the percentage of patients whose therapeutic intervention has led to CR, PR, or SD. Objective response rate (ORR) was defined as the percentage of patients with the best overall response of CR or PR relative to the analysis set. Progression‐free survival (PFS) was defined as the duration from diagnosis or assessment before a treatment regimen to the first day of documented disease progression or death. Overall survival (OS) was defined as the time from diagnosis of advanced disease to death of any cause. Disease‐free survival (DFS) was defined as the time from surgery to recurrence of a tumor or death.

### 2.4 Statistical Analysis

Categorical variables were shown as numbers and percentages (%). Continuous variables with normal distributions were shown as mean ± standard deviation (SD), and those without normal distributions were shown as median (interquartile range, IQR). SPSS software version 23.0 (IBM SPSS Statistics) and GraphPad Prism 8 were used to analyze the results.

## 3 Results

### 3.1 Characteristics of Patients

**FIGURE 1:**
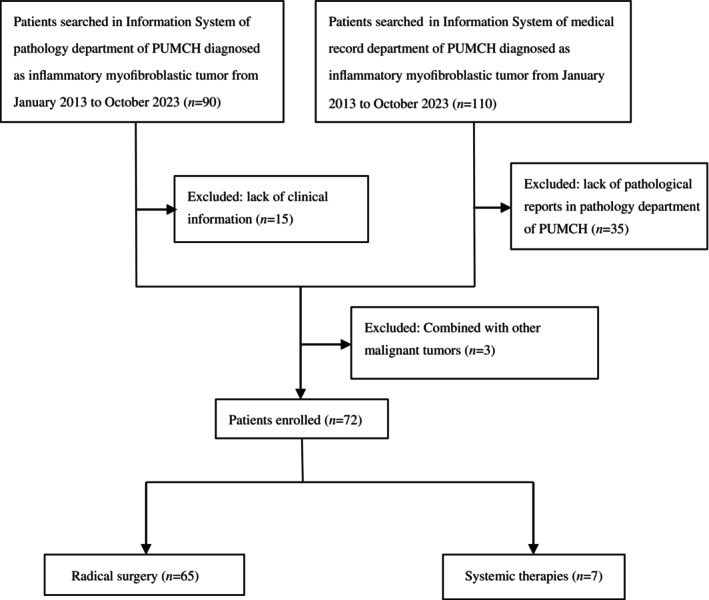
Flow chart of patient selection in this study.

**FIGURE 2:**
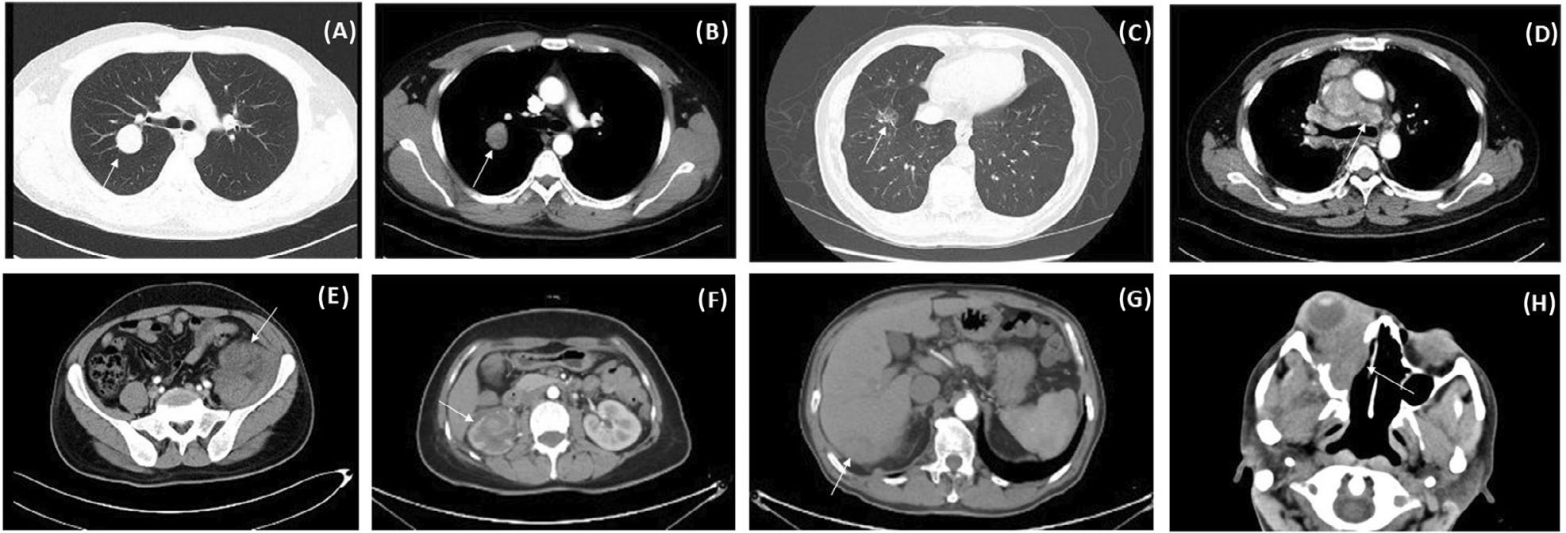
The CT scans of an inflammatory myofibroblastic tumor. (A and B) The CT scan showed a well‐delineated nodule in a patient with a pulmonary inflammatory myofibroblastic tumor. (C) The CT scan showed a ground glass nodule in a patient with a pulmonary inflammatory myofibroblastic tumor. (D) The CT scan showed hilar and mediastinal lymph nodes in a patient with a pulmonary inflammatory myofibroblastic tumor. (E) The contrast‐enhanced CT scan demonstrated heterogeneous enhancement of the left abdominal inflammatory myofibroblastic tumor. (F) The contrast‐enhanced CT scan showed homogeneous enhancement of the right renal inflammatory myofibroblastic tumor. (G) The contrast‐enhanced CT scan demonstrated heterogeneous enhancement right hepatic inflammatory myofibroblastic tumor. (H) The contrast‐enhanced CT scan showed a right nasal sinus inflammatory myofibroblastic tumor involving the orbit.

The process of patient selection is depicted in Figure 1. Nighty patients from the Information System of the pathology department of PUMCH and 110 patients from the Information System of the medical record department of PUMCH were diagnosed with inflammatory myofibroblastic tumors from January 2013 to October 2023. Fifteen records lacked clinical information, 35 records lacked pathological confirmation, and 3 records combined with other malignant tumors. Seventy‐two patients were enrolled in this study. The median follow‐up time was 66.4 months (range, 4.9–128.6 months). The baseline characteristics of IMT patients are shown in Table 1. The median age was 46.5 years (range, 3–80 years). Among them, 52.8% (38/72) of them were males. The most common primary lesions were lung (*n* = 15, 20.8%), intestinal tract (*n* = 8, 11.1%), and abdominal cavity (*n* = 7, 9.7%). Patients with pulmonary IMT presented with cough, fever, and backache. Chest CT scans of most pulmonary IMT patients showed a well‐delineated solitary mass (Figure 2A,B). Only one patient had a ground glass nodule on the CT scan (Figure 2C). One case of advanced pulmonary IMT manifested as hilar and mediastinal lymph node metastasis. (Figure 2D) CT scan of the abdominal cavity, renal, hepatic, and nasal sinus IMT showed a solid mass with heterogeneous or homogeneous enhancement. (Figure 2E–H).

**TABLE 1:** Clinical characteristics of enrolled patients with inflammatory myofibroblastic tumor.

Variables	Values, *n* (%)
Sex	
Male	38 (52.7)
Female	34 (47.2)
Tumor site	
Lung	15 (20.8)
Intestinal tract	8 (11.1)
Abdominal cavity	7 (9.7)
Nasal sinus	5 (6.9)
Muscle of leg	4 (5.5)
Breast	4 (5.5)
Kidney	4 (5.5)
Bladder	3 (4.2)
Retroperitoneum	3 (4.2)
Chest wall	3 (4.2)
Cheek	3 (4.2)
Liver	3 (4.2)
Bone	2 (2.7)
Ureter	1 (1.4)
Throat	1 (1.4)
Vocal cord	1 (1.4)
Brain	1 (1.4)
Testis	1 (1.4)
Vulva	1 (1.4)
Uterus	1 (1.4)
Skin	1 (1.4)
ALK	
ALK positive	30 (41.6)
ALK negative	36 (50.0)
Unknown	6 (8.3)
Extent of tumor	
Resectable	65 (90.3)
Unresectable	7 (9.7)

Abbreviation: ALK, anaplastic lymphoma kinase.

### 3.2 Molecular Characteristics

Sixty‐six patients were tested for ALK expression by IHC. The ALK positivity rate was 45.4% (30/66). NGS was performed in one patient with ALK‐positive, and PLEKHH2‐ALK fusion was detected. NGS was performed in 10 patients with ALK‐negative. Cycle‐dependent kinase 4 (CDK4) amplification, catenin beta 1(CTNNB1) p.T41A mutation, Phosphatase, and Tensin Homolog (PTEN) p.W111R mutation, murine double minute 2(MDM2) amplification, Isocitrate dehydrogenase 1 (IDH1) p.R132H mutation, and platelet‐derived growth factor receptor alpha (PDGFRA) p.S566_E571delinsR mutation were detected.

### 3.3 Treatment of IMT

**FIGURE 3:**
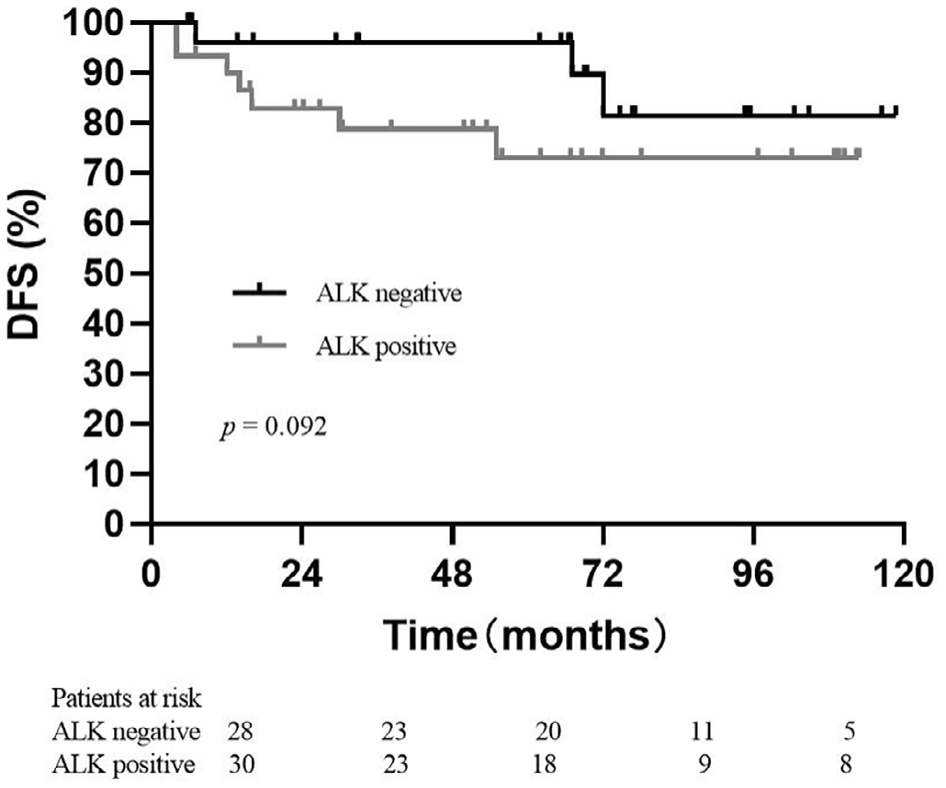
Disease‐free survival in patients with ALK‐negative and ALK‐negative.

Among 72 patients, 65 patients with localized tumors underwent radical surgery. Eleven (16.9%) of these 65 patients had recurrence and/or metastasis after surgery. (Table 2) The median time from surgery to relapse and/or metastasis of these 11 patients was 16 months, ranging from 4 to 72 months. DFS in patients with ALK‐negative had a longer trend than that in patients with ALK‐positive (Figure 3), without a significant difference (*p* = 0.092). Seven patients with locally advanced or metastatic tumors were diagnosed by biopsy and received systemic therapy (Table 3). Thirty patients received systemic treatment, including nonsteroidal anti‐inflammatory drugs (NSAIDs), steroids, chemotherapy, targeted therapy, and immune checkpoint inhibitor. Five patients were treated with pemetrexed plus carboplatin. ORR was 20%, and DCR was 60%. Five patients received prednisone, four of whom had PR. Two patients with ALK positivity were treated with crizotinib, one of whom had PR. Two patients received prednisone, cyclophosphamide, and vincristine, with one PR and one SD. One patient was treated with paclitaxel plus carboplatin and had PR. Only one patient with a high expression of PD‐L1 in the tumor tissue received immune checkpoint inhibitor camrelizumab after chemotherapy and had SD and three‐month PFS.

**TABLE 2:** Characteristics of patients with recurrent inflammatory myofibroblastic tumor after radical surgery.

No.	Sex/age	Primary tumor site	Recurrent tumor site	ALK IHC	Disease‐free survival (months)	Therapies after recurrence	Alive
1	F/39	Nasal sinus	Nasal sinus	+	55	Prednisone+radiotherapy (PR, PFS > 24 months)	Alive
2	F/41	Abdominal cavity	Abdominal cavity	+	16	Crizotinib (PD, PFS 2.0 months)	Death
3	F/3	Brain	Brain	+	4	Surgery+ radiotherapy (PR, PFS 5.0 months)	Death
4	F/19	Skin	Skin	+	4	Radical surgery (PFS > 98 months)	Alive
5	F/59	Breast	Breast	−	67	Radical surgery (PFS > 17 months)	Alive
6	M/50	Lung	Lung	−	72	Pemetrexed+carboplatin (PR, PFS 38 months)	Alive
7	M/43	Lung	Lung	N/A	36	Pemetrexed+carboplatin (SD, PFS 3 months), prednisone (PR, PFS 24 months)	Alive
8	M/41	Muscle of leg	Muscle of leg	+	12	Surgery(CR, PFS 57 months)	Alive
9	M/73	Intestinal tract	Abdominal cavity	−	7	Prednisone, CTX, VCR (SD, PFS 5.0 months)	Death
10	F/51	Ureter	Abdominopelvic cavity	+	14	Surgery (PR, PFS 3 months), crizotinib (PR, PFS > 18 months)	Alive
11	M/69	Lung	Intestine	+	30	Resection of liver metastases (CR, PFS > 6 months)	Alive

Abbreviations: −, negative; +, positive; ALK, anaplastic lymphoma kinase; CR, complete remission; CTX, cyclophosphamide; F, female; IHC, immunohistochemistry; M, male; N/A, not available; PD, progressive disease; PR, partial response; SD, stable disease; VCR, vincristine.

**TABLE 3:** Characteristics of patients with locally advanced and/or metastatic inflammatory myofibroblastic tumor.

	Sex/age	Primary tumor site	Locally advanced or metastatic site	ALK IHC	Systemic therapies	Survival state
1	M/63	Lung	Pleura	−	Prednisone (PD, PFS 1 month); Pemetrexed+carboplatin (PD, PFS 1 month)	Death
2	M/52	Nasal sinus	Locally advanced		Prednisone+CTX + VCR (PR, PFS 7 months)	Alive
3	F/36	Nasal sinus	Locally advanced	−	Prednisone (PR, PFS 5 months)	Alive
4	F/47	Lung	Liver	−	Pemetrexed+carboplatin (SD, 16 months); Paclitaxel+carboplatin (PR, 5 months)	Death
5	M/30	Retroperitoneum	Muscle	+	Ibuprofen (PR,PFS 84 months)	Alive
6	M/49	Abdominal cavity	Bone	−	Pemetrexed+carboplatin (PD, 1 months), camrelizumab (SD, PFS 3 months)	Death
7	M/69	Retroperitoneum	Ureter	−	Prednisone (PR, PFS > 35 months)	Alive

Abbreviations: −, negative; +, positive; ALK, anaplastic lymphoma kinase; CR, complete remission; CTX, cyclophosphamide; F, female; IHC, immunohistochemistry; M, male; N/A, not available; PD, progressive disease.; PR, partial response; SD, stable disease; VCR, vincristine.

## 4 Discussion

As IMT is a rare disease, there were few large sample studies. It was reported that 50%–60% of patients with IMT are children and young adults [5]; however, it can present in older populations. Only 5 patients in our study were younger than 18 years, which was mainly due to the fact that adults are the main patients in PUMCH [5]. The proportion of female‐to‐male in this study was 0.89, which was lower than that in other studies. The ALK positive rate was 45.4% (30/66) in this study, which was close to that reported [6]. Different forms of ALK fusion were reported in IMT, and PLEKHH2‐ALK fusion was detected for the first time in this study [7]. As this patient was not treated with ALK‐TKI, the efficacy of ALK‐TKI for this ALK fusion is uncertain. Besides, this study detected amplification of CDK4 and MDM2, and mutation of CTNNB1, PTEN, IDH1, and PDGFRA. Amplification of CDK4 and MDM2 in IMT was reported [8, 9, 10], which was considered one of the tumorigenic drivers in IMT. Therefore, NGS was suggested to be performed in IMT patients with ALK‐negative. Rearrangement of other genes, such as ROS1, platelet‐derived growth factor receptor beta (PDGFRB), and RET were reported in IMTs with ALK‐negative, which were not detected in this study. Probably because the sample size of the study was not large enough [7].

Most pulmonary IMTs appeared as well‐delineated solid lesions on the CT scan. However, it was found that one patient with pulmonary IMT presented as a ground glass nodule on the CT scan. It showed that the radiologic characteristics of IMTs are heterogeneous.

Surgical resection is the main treatment for localized IMTs. A total of 90.3% (65/72) of the patients had resectable IMT disease and had surgery. Among them, 16.9% (11/65) of patients had relapses or metastases after surgery, which was close to that in another study. DFS in patients with ALK‐positive seemed shorter than that in patients with ALK‐negative, which reminded clinicians of intensive follow‐up for patients with ALK‐positive after the operation. Adjuvant therapy is not regularly recommended for patients with IMT after surgery [11]. ALK tyrosine kinase inhibitor (TKI) alectinib was effective as adjuvant therapy in patients with non‐small cell lung cancer [12], but was not confirmed effective in patients with IMT. Because of the high proportion of ALK‐positive IMT patients, it is worth exploring the efficacy of ALK TKIs as perioperative treatment.

There is no standard chemotherapy regimen for IMTs. A retrospective study indicated that anthracycline‐based and methotrexate plus/minus vinorelbine/vinblastine regimens are very effective in IMT. Pemetrexed plus carboplatin, which is used to treat non‐small cell lung cancer, was the most commonly used chemotherapy regimen in this study [13]. Two patients received pemetrexed plus carboplatin and had quite long PFS. The pemetrexed plus carboplatin regimen could be a treatment option for IMT patients. IMT patients with ALK‐positive are sensitive to ALK TKIs. Only two patients received crizotinib treatment in this study. One patient with ALK‐positive IHC did not respond to crizotinib, while the ALK rearrangement pattern was not known due to insufficient specimen. The ALK inhibitor treatment in IMT is off‐label and not covered by medical insurance, which meads the ALK inhibitor was not available for some patients. It was reported that IMTs showed frequent PD‐L1 expression. However, there are rare reports on the efficacy of ICI in patients with IMT [14]. It was reported that a patient with PD‐L1‐positive nasopharyngeal IMT had a near‐complete remission after 16 cycles of anti‐PD‐1 antibody. In this study, one patient received immunotherapy and had only 3‐month PFS. The efficacy of immunotherapy in treating IMT still needs further exploration [15].

This study had some limitations. First, it was a small sample single‐center retrospective study, which led to selection bias, information bias, and generalizability. Second, the NGS was not conducted in most patients with ALK‐negative, which limited the understanding of the molecular pathology for these patients.

## 5 Conclusions

IMT can occur at any site, and one of the most common sites of IMT is the lung. Surgery is the main treatment for IMT, and postoperative adjuvant therapy for ALK‐positive patients needs to be focused. The molecular testing is essential for all patients diagnosed with IMTs to detect genetic alterations other than ALK. Systemic treatment, including chemotherapy and immunotherapy, needs further research.

## Author Contributions

**Xiaoyan Si** and **Shafei Wu:** contributed to data analysis, statistical analysis, and manuscript preparation. **Ruie Feng:** contributed to pathological data acquisition. **Mengzhao Wang**, **Hanping Wang**, and **Xiaotong Zhang:** contributed to clinical data acquisition. **Xiaoyan Si**, **Li Zhang**, and **Kaifeng Xu:** designed this study. All authors have read and approved the manuscript.

## Conflicts of Interest

The authors declare no conflicts of interest.

## Supporting information


**Table S1.** Genes included in AmoyDx HANDLE Classic Panel.

## Data Availability

The data that support the findings of this study are available from the corresponding author upon reasonable request.
