# Supplementary material for: Clinicopathological Characteristics of Inflammatory Myofibroblastic Tumor: A Single Center Retrospective Cohort Study
Source: Thorac Cancer. 2024 Nov 26;16(1):e15496. doi: 10.1111/1759-7714.15496 (PMC11729751; doi:10.1111/1759-7714.15496)
Supplement: Supplementary file 1 — Table S1. Genes included in AmoyDx HANDLE Classic Panel. [file TCA-16-e15496-s001.docx]

**Supplementary Table S1.** Genes included in AmoyDx® HANDLE Classic Panel.

| AKT1 | FGFR1 | MAP2K1 | PDGFRA |
| --- | --- | --- | --- |
| ALK | FGFR2 | MET | PIK3CA |
| BRAF | FGFR3 | MYC | POLE |
| CDK4 | FGFR4 | NFE2L2 | PTEN |
| CTNNB1 | HRAS | NKX2-1 | RB1 |
| DDR2 | IDH1 | NRAS | RET |
| DPYD | IDH2 | NRG1 | ROS1 |
| EGFR | KEAP1 | NTRK1 | STK11 |
| ERBB2 | KIT | NTRK2 | TP53 |
| ESR1 | KRAS | NTRK3 | UGT1A1 |
